# Abducens Nerve Palsy as a Rare Complication of Herpes Zoster Ophthalmicus: A Case Report

**DOI:** 10.7759/cureus.57506

**Published:** 2024-04-03

**Authors:** Abeer Hisham Sayed, Razan Hassan Gendil, Shamima Mohammed, Laieba Tasneem

**Affiliations:** 1 Neurology, Saudi German Hospital, Dubai, ARE; 2 General Surgery, Soba University Hospital, Khartoum, SDN

**Keywords:** herpes zoster ophthalmicus, abducens nerve palsy, ophthalmoplegia, diplopia, herpes zoster ophthalmicus-related ophthalmoplegia (hzoro)

## Abstract

Herpes zoster ophthalmicus (HZO) is a condition resulting from the reactivation of dormant varicella zoster virus within the sensory nerve ganglion in the ophthalmic branch of the trigeminal nerve. The tell-tale rash along one side of the nerve tract accompanied by pain, a burning sensation, and itching alerts health practitioners on the right path to diagnosis. Conversely, HZO can present with other rarer complications such as intraocular and extraocular manifestations.

This case report deals with a seemingly healthy 45-year-old female who developed left abducens nerve palsy within one week of developing a vesicular rash on the same side. Curiously, those afflicted are usually of an advanced age or suffer from an immunocompromised state; this patient however suffered from no other comorbidities nor did she report having been in contact with anyone of a similar affliction. In this case, the classical treatment regime of antivirals and corticosteroids resulted in the complete resolution of the infection and the return of full ocular function. Being able to recognize and appreciate these typical and atypical signs and symptoms of HZO can aid in the further propagation of good outcomes and timely resolutions.

## Introduction

Herpes zoster ophthalmicus (HZO) represents a distinctive manifestation of varicella-zoster virus (VZV) infection. It is caused by localized reactivation of latent VZV, which typically remains dormant in sensory ganglia following primary infection with chickenpox. The reactivated virus travels along the sensory nerves to reach the ophthalmic branch of the trigeminal nerve, leading to inflammation and tissue damage [1]. Various factors, such as advanced age, immunocompromised states (HIV/AIDS, immunosuppressive therapy), and stress contribute to the reactivation of VZV, emphasizing the multifaceted nature of HZO's etiology [1,2].

The hallmark of HZO is the characteristic vesicular rash that follows the dermatomal distribution of the ophthalmic branch. Patients often present with pain, itching, and a burning sensation preceding the appearance of the rash [3]. Ocular involvement may range from mild conjunctivitis to severe keratitis, and in some instances, acute retinal necrosis [1]. Prompt recognition and intervention are crucial in preventing irreversible ocular damage.

HZO, however, is not limited to a classical presentation. Atypical manifestations can complicate the diagnostic landscape, posing challenges for healthcare providers. These may include neurological complications (cranial nerve palsies, encephalitis) and posterior chamber involvement (retinitis, acute retinal necrosis, or optic neuritis). Other atypical presentations include zoster sine herpete with minimal or no skin involvement [1]. The diversity in clinical presentation emphasizes the necessity for a comprehensive understanding of both typical and atypical manifestations to facilitate accurate diagnosis and appropriate management.

Here we present a case of a young patient with no comorbidities who developed abducens nerve palsy as a complication of HZO.

## Case presentation

**Figure 1:**
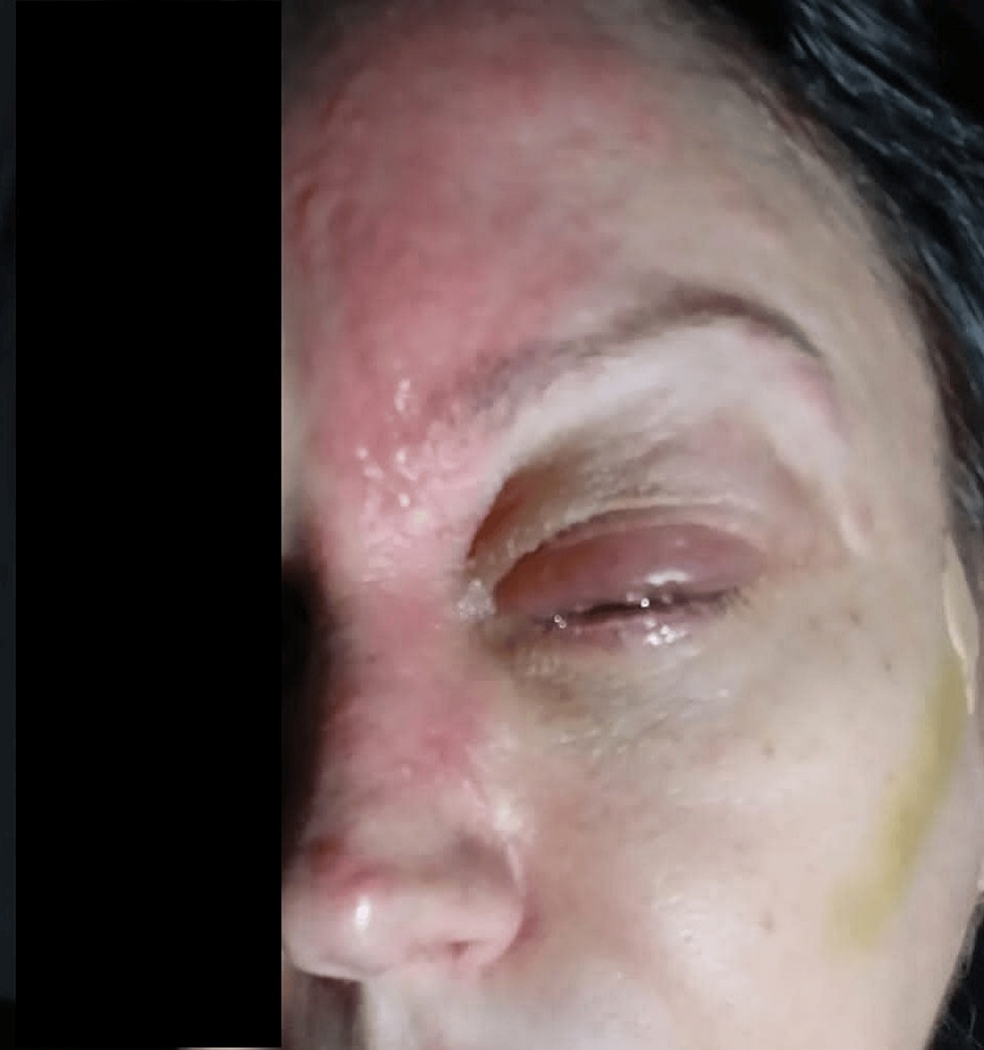
Eyelid edema closing the eye shut. Erythematous skin rash with vesicles extending along the left V1 distribution, with a positive Hutchinson's sign.

The patient was a 45-year-old female with no significant medical history who came to our neurology clinic presenting with a vesicular rash on the upper left side of her face, a worsening headache, and abducens nerve palsy. She was diagnosed eight days prior with HZO at the emergency department of a different hospital. When first examined, the rash covered the dermatome of the ophthalmic division of the trigeminal nerve, and extended to the left ear and part of the scalp, with a positive Hutchinson's sign. Her left eyelids were swollen shut making her eyeball indiscernible (Figure 1). Furthermore, she complained of a gritty burning sensation and watery eyes. The CT scan and basic blood work ordered in the emergency room showed no abnormalities. She was given IV painkillers and was discharged on antibiotics, anti-inflammatories, and a corticosteroid eye gel.

A week later, once the eyelid edema had subsided, the patient noticed that her left pupil had shifted inwards thus she scheduled an appointment to visit an ophthalmologist. The slit lamp examination was normal, the anterior chamber was normal in depth and content, intraocular pressure was within normal limits, the left conjunctiva was hyperemic, the pupil was round and reactive, the lens was clear, and the fundus showed no abnormal vessels and no exudates or hemorrhage. However, there was a blurring of vision and confirmed binocular horizontal diplopia, so they referred her urgently to see a neurologist.

**Figure 2:**
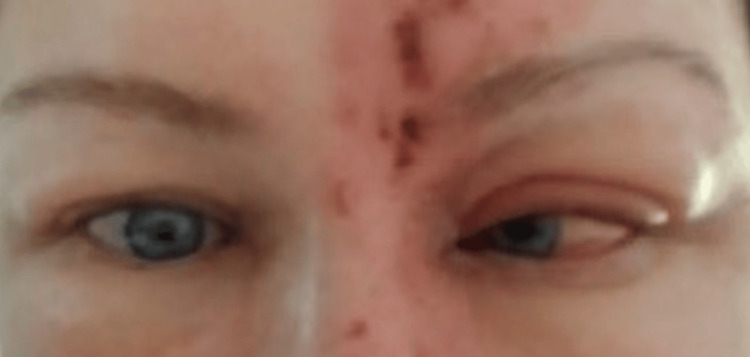
The left eye was fixed medially associated with horizontal diplopia (finding consistent with a diagnosis of abducens nerve palsy).

In our neurology clinic, a basic examination of the neurological system was unremarkable except for the ocular movement examination, which showed a severe limitation in the abduction of the left eye accompanied by horizontal diplopia (Figure 2). An MRI was ordered and it showed no abnormalities. Thus, she was diagnosed as a case of abducens nerve palsy due to HZO.

The patient’s treatment consisted of 1 gram of valacyclovir tablets every eight hours, 5% acyclovir cream every eight hours, along with tobramycin and dexamethasone eye drops every six hours. This regime continued for seven days. In addition, 20 mg prednisone tablets were prescribed twice daily for five days, as well as pregabalin 75 mg three times daily for thirty days. 

On a subsequent follow-up visit a week later, there were clear signs of improvement in her condition, but it was rather slow; thus, she was shifted to IV acyclovir 600 mg three times a day for seven days. Upon completion of her treatment plan, the patient’s headache and herpetic neuritis had improved considerably, the rash had almost disappeared, the vesicles had crusted over, and there was partial improvement of her nerve palsy.

**Figure 3:**
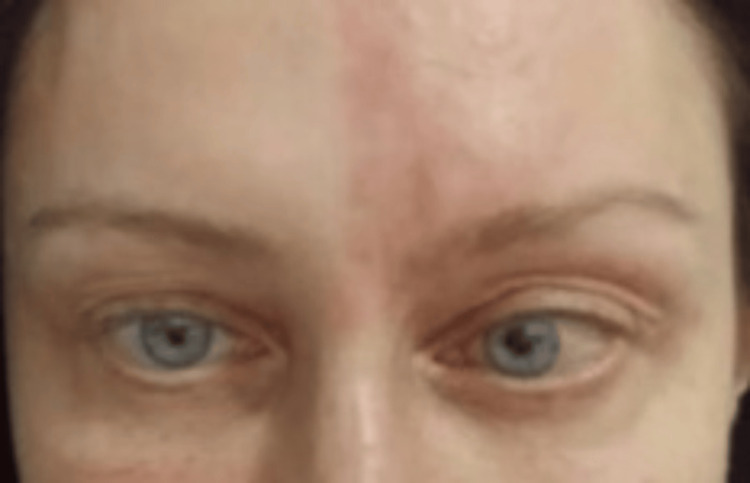
Left eyeball no longer fixated inwardly, and the herpetic rash disappeared indicating resolution.

A month later in her final follow-up, the herpetic rash had cleared up and her skin had returned to normal, and on ocular movement examination, the patient could move her left eye freely in all directions. Thus, there was a complete resolution of her abducens nerve palsy (Figure 3).

## Discussion

Herpes zoster, also known as shingles, is a viral infection that occurs when the VZV reactivates, causing a painful skin rash along distinct nerve routes [2]. It can affect both thoracic (associated with the chest and upper back) and cranial nerves (associated with the head and face).

When the ophthalmic division of the trigeminal nerve is affected, the condition is termed HZO. It manifests as a unilateral vesicular rash and burning pain along the dermatomal distribution, in addition to a variety of ocular manifestations like conjunctivitis, uveitis, keratitis, and retinitis [4]. 

In rare cases, HZO can be complicated by extraocular muscle palsies in 13% of cases [5]. Usually, the oculomotor is the most affected extraocular nerve, followed by the abducens, then the trochlear [5]. It’s very rare for HZO patients to have complete ophthalmoplegia [6]. The usual onset of extraocular palsy is two to four weeks from the rash onset, but it can appear any time before or after this period [6]. Our patient showed signs of abducens nerve palsy within a week from rash onset, which is considered early.

The cause for extraocular palsy is unknown but there are many theories suggested in the literature. Kreibig et al. [7] postulated that the palsy is caused by perivasculitis‑myositis and not a neural pathology. Another theory by Edgerton and Godtfredsen hypothesized that etiology could be through contiguous intracavernous radiculomeningitis [8,9]. Other suggested causes include the direct cytotoxic effect of the virus on surrounding neural tissue, an immunological reaction to the virus by the central nervous system, or occlusive vasculitis [10].

This condition usually affects elderly and immunocompromised patients because it requires the reactivation of the virus, which is normally suppressed in healthy individuals. Therefore, it was rather unusual for our patient, an immunocompetent middle-aged woman with no history of recent illness, to present with this condition accompanied by a rare complication on top of that.

The main treatment of HZO is antiviral therapy, which aims firstly to reduce the impact of ocular manifestations as they can lead to blindness if not dealt with urgently. Secondly, it aims to relieve acute neuritis pain and prevent it from progressing to post-herpetic neuralgia [3]. Corticosteroids can prevent occlusive vasculitis in some cases, but they carry the risk of suppressing the immune system leading to increased viral replication [10]. This issue is mainly a concern in the elderly, so it was safe to commence steroids for our patient.

Regardless of medication use, the palsy associated with HZO is known to have a good prognosis with most cases experiencing spontaneous recovery within six to eight weeks [5].

## Conclusions

This case highlights the importance of promptly anticipating the typical as well as the atypical complications that can arise in an HZO affliction. Regardless of the expected disease progression, rare extraocular palsies such as abducens nerve palsy may occur, and more notably at an earlier than projected time period from rash onset. The multifaceted etiology of HZV and this patient’s age and their lack of any significant comorbidity make this case even more unique. Thus, healthcare providers should take note of this and have a more comprehensive understanding of all the possible presentations of HZO.
